# Real-time pharmacovigilance and cardiac risk communication during the COVID-19 vaccination campaign in Israel

**DOI:** 10.17179/excli2026-9596

**Published:** 2026-09-03

**Authors:** Yaakov Ophir, Yaffa Shir-Raz

**Affiliations:** 1 Ariel University, Ariel, Israel; 2 University of Cambridge, Cambridge, UK; 3 University of Haifa, Haifa, Israel

**Keywords:** COVID-19 vaccination, pharmacovigilance, risk communication, myocarditis, vaccine safety, informed consent

## Abstract

During the early phase of the COVID-19 vaccination campaign, the Israeli Ministry of Health (IMOH) informed international health authorities that it had identified “*a large number of myocarditis and pericarditis cases in young individuals soon after Pfizer COVID-19 vaccine*.” This study discloses and analyzes an internal pharmacovigilance dataset obtained from within the IMOH to examine the case-level record associated with this early safety signal and trace how the signal was subsequently communicated to physicians and the public. The anonymized dataset, provided as Supplementary Data File 1, includes 531 hospitalization records and 67 unique deaths reported to Pfizer between March 2021 and May 2022. Reported events spanned cardiac, neurological, inflammatory, and other serious clinical categories and involved a substantial proportion of younger individuals, with 211 of 530 hospitalization records with valid age data (39.8 %) involving patients under 30 years of age. Myocarditis was recorded in two of the nine unique deaths among individuals under 50 years of age (22.2 %), while myocarditis or pericarditis was identified in 250 of 531 hospitalization records (47.1 0 %), rising to 151 of 211 records involving patients under 30 (71.6 %). Among younger hospitalized patients, 134 of 211 (63.5 %) had no meaningful medical history identified under the study definition; within this subgroup, 99 of 134 (73.9 %) had myocarditis or pericarditis, of whom 90 (90.9 %) were male. The safety signal was communicated to clinicians and the public only after approximately three to four months, primarily in ways that emphasized its rarity, generally mild clinical course, and lower concern relative to COVID-19 outcomes. The accuracy and completeness of this characterization, together with the implications of the case-level patterns and communication timeline documented in this study, are discussed in the broader contexts of pharmacovigilance, risk communication, and informed consent during large-scale public health interventions.

See also the graphical abstract(Fig. 1).

## Introduction

Approximately two months after the launch of the global COVID-19 vaccination campaign, the Israeli Ministry of Health (IMOH) alerted the U.S. Centers for Disease Control and Prevention (CDC, 2023[6]) and the European Medicines Agency (EMA, 2022[11]) about “*a large number of myocarditis and pericarditis cases in young individuals soon after Pfizer COVID-19 vaccine*” (CDC, 2023, p. 668[6]). At that time, Israel was often described as the “world's laboratory” because of its leading vaccination rates and nationwide real-world studies (Birnhack, 2021[4]), and the CDC memoranda released under the Freedom of Information Act marked this correspondence as “High Importance” (CDC, 2023[6]).

What exactly stood behind this early warning of “large numbers” of myocarditis cases? How substantial was this safety signal in reality? And were physicians and the public adequately and promptly informed about it?

In this article, we disclose and analyze an internal IMOH pharmacovigilance dataset obtained from within the Ministry and containing case-level records reported to Pfizer during the COVID-19 vaccination campaign (see the Methods). The objectives of the article are threefold:

1. To make the dataset accessible to the medical and scientific community through an anonymized Supplementary Data File and provide a transparent account of the assembled case-level records of serious adverse events following COVID-19 vaccination.
2. To reconstruct the reporting timeline and evaluate when and how the emerging safety signal and related case information were communicated to health professionals and the public.
3. To situate the findings within pharmacovigilance standards and assess the timeliness, accuracy, and completeness of subsequent public communication about the signal.

## Methods

### Data source and provenance

The internal pharmacovigilance dataset, obtained through a protected source, is provided with this article as Supplementary Data File 1. It comprises two Excel worksheets documenting hospitalizations and deaths following COVID-19 vaccination that were reported by the Israel Ministry of Health (IMOH) to Pfizer during the vaccination campaign. The source worksheets contained detailed case-level information on demographic characteristics, vaccine dose and lot numbers, reported post-vaccination intervals, reasons for hospitalization or circumstances of death, hospitalization status, medical history, clinical narratives, and reporting dates (Table 1(Tab. 1)).

The present analysis used the latest of eight cumulative internal versions made available to the authors through the protected source, covering records reported to Pfizer from March 25, 2021, to May 9, 2022. Comparison of the versions showed that additional cases were incorporated over time; the analyzed version therefore reflects the dataset as of May 2022. The earlier versions were also examined to characterize how the dataset developed and was updated over time.

All eight versions share the same original creation date, March 25, 2021, and identify Noa Cedar as the creator of the original workbook. At the time, Cedar was affiliated with the IMOH Division of Epidemiology and coordinated the monitoring of adverse events occurring in temporal proximity to vaccination. She was also listed as an equal-contribution author of a related nationwide Israeli study of myocarditis following BNT162b2 vaccination (Mevorach et al., 2021[26]). The study's Supplementary Appendix further states that she “performed the active data collection and participated in writing.”

That study described an active myocarditis surveillance system operated by the IMOH during a period that overlapped with the creation and updating of the disclosed workbooks. The convergence of the workbook creator's identity, her documented institutional and research roles, the overlapping surveillance period, and the nature of the information recorded supports linking the workbooks to the IMOH's pharmacovigilance activities. However, neither the article nor its Supplementary Appendix identifies these specific Excel workbooks as a data source for the study. The workbook metadata also do not establish whether Cedar personally collected or entered every record or whether other IMOH personnel contributed to compiling and updating the files.

A distinct structural change appears in the Hospitalizations worksheet among records assigned a reporting date to Pfizer of June 10, 2021. After a largely consecutive case-number sequence through case no. 221, a separate block of 34 records appears in Excel rows 223-256. All 34 concern myocarditis, perimyocarditis, or myopericarditis, retain nonconsecutive source case numbers ranging from 222 to 310, and share the same reporting date to Pfizer. After this block, the broader case-number sequence resumes at no. 311. The cardiac records therefore appear to have been incorporated as a distinct set and may have been drawn from a broader numbered source rather than entered as part of the preceding consecutive sequence. The workbooks, however, do not identify the source of these records or the basis for their inclusion.

Comparison of the successive versions indicates that the workbook functioned as an evolving working dataset. In addition to the incorporation of new cases, later versions added clinical information to existing records, revised diagnoses and reporting dates, and omitted some records that had appeared in earlier versions. For example, an initial diagnosis of ARDS in one record was subsequently changed to acute eosinophilic pneumonia after additional clinical information was added. In another record, the diagnosis was changed from pericarditis to myocarditis and later changed back to pericarditis. Between the October and December versions, the reporting date to Pfizer was changed from October 11 to October 7, 2021, in 46 records, indicating a coordinated revision across these records. In addition, one death report present in the October version was no longer present in the December or any subsequent version. The workbooks contain no formal audit trail identifying who made these changes or the reasons for them.

During data-quality review, we identified an Excel comment authored by Cedar and attached to case no. 343, stating, in translation from Hebrew, “Duplicate of case no. 340; can be deleted.” The two records matched with respect to initials, age, sex, vaccine dose number, lot number, interval between vaccination and event onset, and reporting date to Pfizer. Case no. 343 was therefore excluded from all analyses. To preserve source fidelity, the record was retained in Supplementary Data File 1, and the original workbooks were preserved without modification. Following this exclusion, the analytical dataset contained 531 hospitalization reports.

The workbooks were not accompanied by methodological documentation or a data dictionary. Accordingly, the available materials do not permit us to independently establish how all cases were identified, selected, or entered; who entered each record; whether the workbooks represent a complete export or a subset curated for reporting to Pfizer; what additional clinical-validation or quality-control procedures were performed; or the form in which the recorded information was transmitted to Pfizer. The implications of these uncertainties are addressed further in the Limitations section.

Taken together, the workbook metadata, institutional attribution, detailed case-level structure, cumulative updating across versions, and explicit reporting dates to Pfizer support characterizing the files as an operational IMOH pharmacovigilance dataset used to document hospitalizations and deaths reported to Pfizer following COVID-19 vaccination. This characterization does not establish that the workbooks captured all relevant events, that every reported diagnosis was clinically validated, or that the reported events were causally related to vaccination. Their inclusion does, however, indicate that the events were regarded as sufficiently temporally or clinically relevant to vaccination to warrant pharmacovigilance documentation and transmission to the manufacturer for assessment.

### Data preparation and classification

To facilitate independent inspection and reproduction of the analyses, Supplementary Data File 1 retains the original, predominantly Hebrew, source fields and includes bilingual column headings, a README and codebook, standardized numeric variables, and record-specific data-quality and coding notes. Patient initials, medical-history free text, and clinical narratives were removed to protect confidentiality and are marked “Anonymized.” The derived analytical variables were coded from the complete source file before these fields were removed.

Source case numbers were retained as recorded and were not assumed to constitute unique identifiers. The Hospitalizations worksheet contained 532 source rows. An original Excel comment attached to case 343, authored by Noa Cedar, states that the record is a duplicate of case 340 and may be deleted. To preserve fidelity to the source file, both records were retained in Supplementary Data File 1; however, case 343 was excluded from all analyses, leaving 531 hospitalization records in the analytical dataset. Apart from this source-identified duplicate, each remaining spreadsheet row was treated as a distinct source record. The two worksheets were also cross-compared to identify records referring to the same death before calculating the number of unique deaths.

Because myocarditis and pericarditis constituted the primary safety signal communicated by the IMOH to international regulators, we conducted a focused classification of these diagnoses within both worksheets. Cases were identified through explicit Hebrew or English references to myocarditis, pericarditis, myopericarditis, or perimyocarditis, including clear spelling variants, in the reason-for-hospitalization, diagnosis, or circumstances-of-death fields. The original clinical narratives were also reviewed to identify cases in which an explicit diagnosis appeared only in the narrative. The primary analysis included diagnoses identified either in the designated fields or in the clinical narratives, whereas a sensitivity analysis was restricted to diagnoses appearing in the designated diagnosis or outcome fields. The corresponding numeric coding is documented in Supplementary Data File 1.

For the hospitalization records, meaningful_medical_history was coded as 1 when meaningful medical history was documented and 0 when no meaningful medical history was identified in the available record. The latter category included records explicitly indicating no medical history, records in which medical history was unknown or not reported, and records describing only minor conditions considered unlikely to account for the serious reported event, such as ADHD, anxiety, or smoking.

The original free-text hospitalization-status field was standardized as patient_status: 0 = discharged, 1 = hospitalized, 2 = died, and 9 = unknown, unclear, or under evaluation. In one record, the patient was initially reported as discharged but was subsequently reported to have died; this record was coded according to the latest documented outcome.

The original time fields contained a mixture of numeric values and Hebrew free-text descriptions. A numeric variable, time_from_vaccination_days, was therefore created. Intervals reported in minutes or as less than 24 hours were coded as 0 days. Longer intervals expressed in hours were converted to completed 24-hour periods (e.g., 35 hours was coded as 1 day and 60 hours as 2 days); weeks were multiplied by seven; and one month was coded as 30 days.

Potentially ambiguous, implausible, or internally inconsistent values were retained as reported and documented in data_notes rather than silently corrected. One source age value of 665 years was retained in the supplementary dataset but treated as missing in age-based analyses.

### Statistical and contextual analyses

The analyses consisted primarily of descriptive statistics and chronological tabulation of cases reported to Pfizer. Categorical variables are presented as frequencies and percentages, with 95 % Wilson score confidence intervals for the principal proportions. To further assess the internal robustness of the descriptive patterns, exploratory subgroup comparisons were conducted using two-sided Fisher's exact tests and are reported as odds ratios with 95 % confidence intervals. A sensitivity analysis examined whether the myocarditis/pericarditis estimates changed when classification was restricted to diagnoses recorded in the designated diagnosis fields.

In addition, the case-level dataset was cross-referenced with contemporaneous regulatory communications and published studies to reconstruct how the myocarditis/pericarditis safety signal was identified, interpreted, and communicated. Supplementary Table 1 provides additional contextual evidence on the clinical outcomes and pathophysiological mechanisms reported in the literature on COVID-19 vaccine-associated myocarditis. These contextual analyses were descriptive and were not subjected to inferential statistical testing.

Importantly, the analyses are not intended to estimate incidence rates, population-level risk, vaccine effectiveness, or causality. Rather, given the structure and limitations of the internal IMOH dataset, their purpose is to describe the safety-related information contained in the reported cases, examine the internal consistency of the principal descriptive patterns, and assess how the emerging information was communicated to physicians and the public.

## Results

The Deaths worksheet contained 62 records. The median reported interval from vaccination to death was 3 days (IQR, 1-7 days; *n* = 62). The Hospitalizations worksheet contained 531 analytical records (after exclusion of the source-identified duplicate; see Methods), with a median reported post-vaccination interval of 5 days (IQR, 3-15 days; *n* = 529); two records lacked a reported interval. Six hospitalization records indicated that the patient died. Cross-comparison of the two worksheets identified one individual (a 22-year-old woman) who appeared in both worksheets. Counting this individual only once yielded a total of 67 unique deaths across the dataset.

The final workbook contained 36 distinct dates in the “Date Reported to Pfizer” field, spanning March 25, 2021 to May 9, 2022 (Table 2(Tab. 2)). The earliest recorded reporting date, March 25, 2021, included 157 hospitalization records and 46 death records.

Taken together, (1) the detailed case-level clinical documentation, (2) the generally short intervals between vaccination and adverse events, (3) the formal reporting to Pfizer, and (4) the fact that the dataset was obtained from within the Ministry of Health support interpreting the spreadsheet as a genuine pharmacovigilance record of cases considered sufficiently relevant to vaccination.

### Age distribution and medical history

The dataset included a notable proportion of young individuals. Nine of the 67 unique deaths involved individuals under 50 years of age (13.4 %; 95 % CI, 7.2 %-23.6 %): eight were recorded in the Deaths worksheet and one additional unique death was identified in the Hospitalizations worksheet. Among the 530 hospitalization records with valid age data, 211 involved individuals under 30 years of age (39.8 %; 95 % CI, 35.7 %-44.0 %). Of these, 134 records (63.5 %; 95 % CI, 56.8 %-69.7 %) had no meaningful medical history identified under the study definition.

### Serious adverse events

The hospitalization records documented a broad spectrum of serious clinical events. These included **cardiac events** (e.g., chest pain, arrhythmias, syncope, heart failure, myocarditis, and pericarditis); **neurological disorders** (e.g., seizures, stroke, encephalitis, Guillain-Barré syndrome, and cranial nerve palsies); **allergic reactions** (e.g., urticaria, angioedema, and suspected anaphylaxis); **inflammatory and hematological syndromes** (e.g., multisystem inflammatory syndrome in adults and immune thrombocytopenic purpura); and other **serious conditions**, including acute kidney injury and sudden sensorineural hearing loss.

For the present analysis, however, we focus specifically on myocarditis and pericarditis - the principal safety signal formally reported by the IMOH to the CDC and EMA, as described in the Introduction.

### Myocarditis and pericarditis

Myocarditis was recorded in the circumstances-of-death field for two of the nine unique deaths among individuals under 50 years of age (22.2 %; 95 % CI, 6.3 %-54.7 %) - a 22-year-old woman and a 47-year-old man. The additional unique death under age 50 identified in the Hospitalizations worksheet involved a 35-year-old man whose hospitalization diagnosis was worsening heart failure; myocarditis was not recorded.

Among the 531 hospitalization records, 250 (47.1 %; 95 % CI, 42.9 %-51.3 %) were classified as involving myocarditis or pericarditis (Table 3(Tab. 3)). The concentration was more pronounced among younger patients: 151 of the 211 hospitalization records involving individuals under 30 years of age (71.6 %; 95 % CI, 65.1 %-77.2 %) involved myocarditis or pericarditis, including 48 patients younger than 18 years.

Among these 151 younger patients with myocarditis or pericarditis, 99 (65.6 %; 95 % CI, 57.7 %-72.7 %) had no meaningful medical history identified under the study definition. Viewed from the complementary direction, 99 of the 134 patients under 30 with no meaningful medical history identified (73.9 %; 95 % CI, 65.9 %-80.6 %) had myocarditis or pericarditis, and 90 of these 99 patients (90.9 %; 95 % CI, 83.6 %-95.1 %) were male. Selected clinical narratives in Table 3(Tab. 3) further illustrate cases of acute cardiac inflammation developing within days to weeks of vaccination.

### Exploratory subgroup comparisons and sensitivity analysis

Exploratory subgroup comparisons showed that myocarditis or pericarditis was identified in 151 of 211 patients under 30 years of age (71.6 %), compared with 99 of 319 patients aged 30 years or older (31.0 %; OR, 5.59; 95 % CI, 3.82-8.19; p < 0.001). Among patients under 30, myocarditis or pericarditis was identified in 136 of 164 males (82.9 %), compared with 15 of 47 females (31.9 %; OR, 10.36; 95 % CI, 4.96-21.63; p < 0.001). In contrast, the proportion did not differ significantly between patients under 30 without meaningful medical history (99/134, 73.9 %) and those with a meaningful medical history (52/77, 67.5 %; OR, 1.36; 95 % CI, 0.74-2.51; p = 0.345).

In a sensitivity analysis, classification was restricted to cases in which myocarditis or pericarditis was recorded in the designated diagnosis or reason-for-hospitalization field, thereby excluding two cases identified only in the original clinical narratives. Under this more restrictive definition, 248 of 531 hospitalization records (46.7 %; 95 % CI, 42.5 %-51.0 %) and 149 of 211 records involving patients under 30 (70.6 %; 95 % CI, 64.1 %-76.3 %) involved myocarditis or pericarditis. Among the 134 patients under 30 for whom no meaningful medical history was identified, 97 met the restrictive definition (72.4 %; 95 % CI, 64.3 %-79.3 %). Thus, restricting the classification to the designated diagnostic field produced only minor changes in the principal descriptive findings.

### Timeline of the safety signal communication

In the following section, we present a chronological mapping of key communications regarding the myocarditis safety signal, focusing mainly on 2021 - the first year of the vaccination campaign (Table 4(Tab. 4); References in Table 4: AHA, 2021[1]; Barda et al., 2021[3]; CDC, 2023[6]; Chua et al., 2022[8]; EMA, 2022[11]; FDA, 2020[13]; Gargano et al., 2021[17]; Mevorach et al., 2021[26]; Vogel and Couzin-Frankel, 2021[38]; Witberg et al., 2021[39]). This timeline illustrates how the signal was identified, reported, and framed by health authorities and in the scientific literature. To minimize interpretive bias, the table relies primarily on original verbatim quotations. Nevertheless, the selection of quotations may entail some degree of unintentional bias, and we have made every effort to present the material as faithfully and transparently as possible.

As illustrated in Table 4(Tab. 4), throughout 2021 information regarding myocarditis in the context of COVID-19 vaccination was communicated to the public only after considerable delay. When eventually addressed, this communication typically characterized myocarditis and pericarditis cases as rare, mild, and transient, while emphasizing that the dangers of COVID-19 outweighed those associated with vaccination.

## Discussion

The findings from this study suggest that an early pattern of reported cardiac adverse events following COVID-19 vaccination was communicated to clinicians and the public only after a delay of approximately three to four months, and was then described as “rare,” “mild,” and generally less concerning than the risks posed by COVID-19. The newly disclosed IMOH dataset, provided with this article in anonymized form as the Supplementary Data File, documents 531 hospitalizations and 67 unique deaths, with generally short reported intervals following vaccination. Reports to Pfizer, which began no later than March 25, 2021, included a notable proportion of young individuals, including many for whom no meaningful medical history was identified. Nearly half of all hospitalization records involved myocarditis or pericarditis, rising to over 70 % among those under 30, the large majority of whom were male. In what follows, we interpret these findings within the broader context of pharmacovigilance standards, medical ethics, and contemporary research on vaccine-associated myocarditis, as reflected in the accumulating clinical and mechanistic evidence summarized in Supplementary Table 1.

### Disproportionate patterns in pharmacovigilance

A central question arising from the disclosed data is whether the observed adverse events represent a genuine safety signal. Although the dataset is observational and lacks background rates (see also the Limitations section), pharmacovigilance does not rely solely on incidence estimates. Notably, contemporaneous communications indicate that officials within the Israeli Ministry of Health, who held the relevant data, interpreted the emerging pattern of cardiac events as involving “*a large number of myocarditis and pericarditis*” (see Introduction). This real-time interpretation is consistent with a key principle in signal detection: *disproportionality*, whereby a specific adverse event becomes unusually prominent within a product's safety profile.

Regulatory guidance recognizes disproportionate reporting as one of several elements that may contribute to signal detection and validation. The EMA's Good Pharmacovigilance Practices lists “disproportionality of reporting, if applicable,” alongside factors such as the number and quality of cases, temporal consistency, patient exposure, and clinical plausibility (EMA, 2017[12]). Quantitative methods such as the Reporting Odds Ratio (ROR) and Proportional Reporting Ratio (PRR) operationalize this principle by comparing the reporting of a specific product-event combination with a reference reporting distribution (CIOMS, 2010[9]; Cutroneo et al., 2024[10]). The MHRA likewise applies quantitative signal criteria, although its cited 8 % threshold concerns a specific change-in-frequency rule for established drug-event combinations rather than a general threshold based solely on an event's proportion within a product's reports (MHRA, 2023[27]). Because the present dataset lacks an appropriate reporting comparator and may represent a selected operational subset, it does not permit formal disproportionality analysis. The observed concentration should therefore be interpreted as a marked within-dataset pattern warranting qualitative signal consideration, rather than as a quantitative disproportionality statistic.

Against this background, the internal dataset shows the following distribution of reported events:

- 47.1 % of all hospitalization records involved myocarditis or pericarditis.
- Myocarditis was recorded in 22.2 % of the unique deaths among individuals under age 50.
- 71.6 % of hospitalization records involving individuals under 30 involved myocarditis or pericarditis.
- Among these younger patients with myocarditis or pericarditis, 65.6 % had no meaningful medical history identified under the study definition.
- Among patients under 30 for whom no meaningful medical history was identified, 73.9 % had myocarditis or pericarditis; 90.9 % of these patients were male.

This distribution suggests a pronounced imbalance in the reported events *within the dataset*: compared to the broad range of adverse events reported, myocarditis, pericarditis, and deaths in young individuals appear in relatively prominent proportions. At the same time, an external perspective may further contextualize these findings: while isolated cases of myocarditis or sudden death can occur in young people, the repeated observation of such outcomes within a limited set of a few hundred suspected vaccine-related reports may warrant careful consideration.

Historical precedent illustrates that a rare but credible vaccine-safety signal may warrant precautionary regulatory action even before all uncertainties have been resolved. Following epidemiological evidence linking Pandemrix to an increased risk of narcolepsy among children and adolescents, the EMA recommended in July 2011 that its use in persons under 20 years of age be restricted to situations in which seasonal trivalent influenza vaccines were unavailable, while maintaining that its overall benefit-risk balance remained positive (Jeong et al., 2025[20]; Schnirring, 2011[34]). This precedent underscores the importance of timely investigation and communication of emerging safety signals, but it does not provide a directly comparable quantitative benchmark for the magnitude of the pattern observed in the present dataset.

### Timeline of cardiac risk communication

Despite the potential importance of these findings, their communication to physicians and the public was delayed by approximately three to four months (in contrast to the FDA's EUA requirement for transparent reporting). Based on the available documentation (Table 4(Tab. 4)), the following timeline can be established:

1. **On February 28, 2021,** Israel alerted the EMA and CDC to ~40 myocarditis cases.
2. **On March 10, 2021,** CDC internal communications appeared to provide a more limited characterization of Israel's warning, by noting that Israel had not provided details and citing only 27 VAERS cases.
3. **On March 25, 2021,** Israel had already notified Pfizer of 46 deaths and 157 hospitalizations, including 65 cases of myocarditis/pericarditis.
4. **On May 28, 2021,** CDC instructed clinicians to report all myocarditis/pericarditis cases after vaccination to VAERS.
5. **On June 25, 2021,** the FDA modified the EUA fact sheets to include information on myocarditis, and CDC produced educational materials.
6. **On July 6, 2021,** CDC publicly acknowledged the risk in *MMWR*, but reassured the public that cases were generally transient and that the benefits of vaccination outweighed the risks.
7. **Between August 25 and October 6, 2021,** the *New England Journal of Medicine* published several Israeli studies documenting an elevated risk of myocarditis after BNT162b2 vaccination (Barda et al., 2021[3]; Mevorach et al., 2021[26]; Witberg et al., 2021[39]). Among these studies, Mevorach et al. reported the most pronounced subgroup-specific risk estimate: after the second dose, definite or probable myocarditis occurred in approximately 1 in 26,000 male recipients and 1 in 218,000 female recipients overall, while the highest rate ratio was observed among males aged 16-19 years, corresponding to approximately 1 case per 6,637 recipients (Mevorach et al., 2021[26]).

Taken together, this chronology reveals three successive layers of risk communication following the first documented warning in late February: communication to clinicians after approximately three months, formal regulatory acknowledgment after approximately four months, and fuller peer-reviewed characterization in the scientific literature approximately six to seven months later. Importantly, the delays in the first two layers may have had implications for clinical awareness and informed decision-making. Clinicians who were unaware of the emerging safety signal may not have recognized or managed relevant cases in a timely manner, and patients may not have been in a position to provide fully informed consent for an EUA-authorized medical intervention whose safety profile was still under active investigation.

### Beyond myocarditis: The broader safety record

Among the peer-reviewed publications that formed the final stage of this chronology, the national study by Mevorach et al. (2021[26]) provides the most directly relevant comparison with the present analysis, because the two studies likely drew on substantially overlapping, though non-identical, case material. As noted in the Methods, the creator of the present workbook, Noa Cedar, was an equal-contribution author credited with performing the active data collection for the national study, and both datasets include the distinctive fatal case of a 22-year-old woman with fulminant myocarditis. Their purposes, however, differed: Mevorach et al. used national surveillance data and formal clinical adjudication to estimate myocarditis risk, whereas the present study examines the broader operational record of hospitalizations and deaths reported to Pfizer.

This difference in purpose also produced a narrower analytical field of view. Restricting the national study to clinically adjudicated myocarditis enabled population-based risk estimates, but pericarditis without documented myocardial involvement, events outside the prespecified temporal windows, and non-myocarditis hospitalizations and deaths fell outside its principal analysis. Using July 25, 2021, as a conservative cutoff, the present dataset contained 26 pericarditis-only hospitalization reports, 131 hospitalizations involving other clinical events, and 54 death reports, 52 of which did not record myocarditis (later workbook versions contained 35 additional reports meeting the same dose and post-vaccination interval criteria, but it could not be determined whether the underlying events occurred before or after the July 25 reporting cutoff).

The quantitative consequence of this narrower clinical lens can be illustrated while retaining the follow-up framework used by Mevorach et al. Within the same sex-specific 21-day window after the second dose, the operational dataset contained 52 additional reports among males and 36 among females, comprising pericarditis-only cases, other hospitalizations, and non-myocarditis deaths. Mechanically adding these reports to the published myocarditis numerators would increase the crude composite reporting frequency from 3.83 to 5.90 per 100,000 male recipients (approximately 1 in 26,000 to 1 in 16,900) and from 0.46 to 1.83 per 100,000 female recipients (approximately 1 in 218,000 to 1 in 54,600).

These deliberately conservative figures are not intended to provide revised incidence estimates or measures of causal risk. The case pools were not identical, the completeness and selection criteria of the operational dataset are unknown, the additional outcomes were not clinically adjudicated or causally assessed in a comparable manner, and further overlap between records cannot be excluded. Rather, this deliberately constrained calculation illustrates that even within the national study's temporal framework, the serious reported-event burden was substantially broader than the myocarditis endpoint alone captured. The present study therefore extends the national myocarditis analysis by placing its adjudicated clinical signal within a broader operational context, tracking the timing of the risk communication, and examining whether the resulting characterization of that risk's magnitude and its relation to COVID-19 outcomes was complete and consistent with the wider scientific literature (see below).

### Beyond “rare and mild”: Cardiac risk in the broader scientific record

The scientific literature that informed the communication of myocarditis and pericarditis risk during the COVID-19 vaccination campaign is heterogeneous (Table 4(Tab. 4)). The present discussion does not aim to provide a comprehensive review of this literature, but rather to examine selected, widely cited studies that shaped the prevailing characterization of the risk across two related dimensions: its magnitude and its comparison with the risks associated with COVID-19.

#### 1. The magnitude of the risk

- **A notable pattern.** Although vaccine-associated myocarditis and pericarditis were commonly characterized as rare, the prominence of these cases in the internal spreadsheet and the incidence estimates reported in several independent studies, particularly among young males, suggest that this characterization may not have fully conveyed the magnitude of the signal in specific populations. For example, the Hong Kong study (Chua et al., 2022[8]) reported a rate of approximately 1 in 2,679 male adolescents after the second dose, a notable frequency in the context of a preventive intervention administered to otherwise healthy individuals.
- More recent studies further suggest that the spectrum of post-vaccination cardiac findings may extend beyond cases captured through hospitalization-based surveillance (Supplementary Table 1). For instance, a study of hospital employees detected elevations in markers of myocardial injury in approximately 2.8 % of participants following mRNA-1273 booster vaccination (Buergin et al., 2023[5]). Findings from other surveillance-based and cohort studies similarly indicate varying levels and manifestations of cardiac involvement following vaccination (Mansanguan et al., 2022[25]; Ophir et al., 2025[30]).
- **Potential underestimation.** The reported figures may underestimate the frequency and clinical spectrum of post-vaccination cardiac events. During Israel's first vaccination year, no robust adverse-event reporting system was in place. More generally, underreporting is a well-recognized limitation of spontaneous and semi-structured pharmacovigilance systems, although its extent varies substantially according to the type and severity of the event, reporter awareness, and the design of the reporting system (Kessler, 1993[22]; Kostoff et al., 2020[23]; Lazarus, 2010[24]). Real-time reporting may therefore have been more likely to capture severe and temporally proximate presentations.
- Moreover, the internal dataset analyzed in the present study is limited by design to hospitalizations and deaths and thus does not capture subclinical myocardial injury or milder presentations that did not result in hospitalization (e.g., Ophir et al., 2025[29]). It may also have missed atypical or delayed-onset presentations, which can still result in debilitating or fatal outcomes (Cho et al., 2023[7]). Recent studies suggest that the broader spectrum of cardiac findings includes both subclinical myocardial injury detected through biomarker screening and persistent imaging abnormalities among patients with clinically diagnosed vaccine-associated myocarditis or myopericarditis (Buergin et al., 2023[5]; Mansanguan et al., 2022[25]; Schauer et al., 2022[33]; Supplementary Table 1).
- **Short follow-up periods.** Early studies relied on relatively narrow observation windows. For example, the Israeli study by Barda et al. (2021[3]) used a follow-up framework designed to provide approximately 21 days of observation after each vaccine dose, which may have failed to capture some later-onset cases. Such cases are evident in the internal spreadsheet, in which 12 % of myocarditis/pericarditis reports occurred more than 21 days after vaccination, and in later studies using extended follow-up periods (Supplementary Table 1).
- Longer follow-up may also reveal clinically important outcomes that are not apparent during the acute phase. A large national cohort study with a mean follow-up of approximately 10 years found that patients with a history of acute myocarditis faced increased risks of life-threatening ventricular tachycardia, cardioverter-defibrillator implantation, and cardiovascular mortality (Te et al., 2017[36]). Although this study was unrelated to COVID-19 vaccination and cannot establish that vaccine-associated myocarditis carries the same long-term prognosis, it illustrates why conclusions that myocarditis is uniformly transient or clinically inconsequential cannot be based on short-term follow-up alone.

#### 2. The comparison to COVID-19 outcomes

While direct comparison with background incidence rates or with unvaccinated populations is not feasible within the present dataset, selected cohort and observational studies may provide contextual reference points for interpreting these patterns. At the same time, such comparisons should be interpreted with caution, given the methodological limitations inherent in observational designs.

- **Unreliable observational comparisons.** In the absence of a defined reference population or background incidence rates within the present dataset, and without direct evidence from randomized controlled trials (RCTs), evaluations of vaccine- and disease-related outcomes often rely on observational studies, which are subject to well-known biases such as the healthy vaccinee effect (Fürst et al., 2024[14][15]). Notably, when examining the Pfizer clinical trial data, where randomization allows a more controlled comparison between groups, the difference in severe COVID-19 cases was small (on the order of a few cases), whereas serious adverse events were reported more frequently in the vaccine group than in the placebo group (Ophir, 2026[28]; Zakov, 2022[40]). These findings suggest that, when comparisons are made under more controlled conditions, the balance between benefits and harms may be less favorable than implied by observational comparisons.
- **Low baseline disease risk.** The assumption that COVID-19 posed substantial cardiac risks to young males remains debated. A large Israeli cohort study of ~800,000 unvaccinated individuals (conducted before the vaccination campaign) found no difference in myocarditis rates between infected and uninfected persons (Tuvali et al., 2022[37]). Moreover, the demographic group identified as most vulnerable to vaccine-associated myocarditis (young males) had a very low baseline risk from COVID-19 (Andrews et al., 2026[2]). A study analyzing over one million medical records of British children and adolescents reported that “*Across all analyses there were no COVID-19-related deaths, and fewer than seven COVID-19-related critical care admissions. Myocarditis and pericarditis were documented only in the vaccinated groups*” (Andrews et al., 2026[2]).
- **Misapplied risk framework.** Importantly, comparing disease-related and vaccine-related risks is meaningful primarily when vaccines prevent infection, allowing researchers to weigh a certain, one-time vaccine exposure against the uncertain probability of contracting the disease. COVID-19 vaccines did not provide sterilizing immunity (Ophir et al., 2023[32]; Ophir et al., 2025[31]), leaving recipients exposed to both vaccine-related and disease-related risks (even if the latter were somewhat reduced). Under these conditions, the observational comparison becomes potentially misleading.
- **Misclassification of exposure.** Observational studies frequently fail to distinguish between vaccinated and unvaccinated individuals within “COVID-19 infection” groups. For example, a large Nordic cohort study stated that “*if an individual had received an mRNA vaccine and had a positive PCR test result for SARS-CoV-2 infection within 28 days, the latest exposure defined the type of myocarditis*” (Husby et al., 2023[18]). Under such a rule, myocarditis cases potentially triggered by vaccination or by vaccine-infection interactions (including possible Vaccine-Enhanced Disease mechanisms) could be erroneously attributed to COVID-19 infection. This type of misclassification may also bias pooled estimates in meta-analyses (Ishisaka et al., 2024[19]).
- **Cumulative exposure.** Comparisons often overlook the additive risks of repeated dosing (two primary doses followed by periodic boosters) (Gao et al., 2023[16]). This cumulative risk is further underscored by emerging evidence of long-term myocarditis sequelae, as reported in recent clinical and mechanistic studies (Supplementary Table 1) - evidence that is likewise often not fully accounted for in prevailing risk-benefit discourse.
- **Alternative findings.** Finally, despite the aforementioned disparities that tend to inflate infection-related risk estimates, a Nordic cohort study of 23 million residents found only 1.37 excess myocarditis cases per 100,000 after COVID-19 infection among males aged 16-24, compared with 5.55-18.39 per 100,000 after a second vaccine dose (Karlstad et al., 2022[21]). Similar estimates were reported for pericarditis, indicating a higher reported vaccine-associated risk.

Taken together, these considerations suggest that characterizing vaccine-associated myocarditis as rare, mild, transient, or less concerning than COVID-19 risks, may have been incomplete or potentially inaccurate, with possible implications for clinical awareness and informed consent.

## Limitations

The case-level granularity of the IMOH dataset represents a major strength of this study, yet its nature also entails several limitations.

First, as a descriptive case-report dataset, it cannot establish causal relationships between vaccination and reported outcomes. Moreover, the dataset is not linked to a defined population denominator, and the available documentation does not establish that the cases were collected through a standardized, population-based surveillance protocol. Accordingly, it does not permit estimation of incidence rates or population-level risk, nor direct comparison with background incidence rates or with rates observed in unvaccinated or infected populations. The proportions, odds ratios, and confidence intervals reported in this study describe patterns within the assembled case reports and should not be interpreted as estimates of relative or absolute risk in the vaccinated population.

Second, the provenance, construction, and completeness of the internal dataset cannot be fully established, as the criteria and processes by which cases were identified, compiled, and transmitted are not fully documented. The available materials do not identify who entered or revised individual records, what clinical-validation or quality-control procedures were applied, or the form in which the recorded information was transmitted to Pfizer. It therefore cannot be determined whether the dataset represents all relevant reports or a selected subset, raising the possibility of both selection and reporting bias. This concern is reinforced by a May 2024 report by the State Comptroller, which found that “the Ministry of Health's computerized systems did not record about 82% of the information of about 354,200 reports that were forwarded to it by medical sources” (State Comptroller of Israel, 2024[35]). Although this finding does not establish the extent of under-recording in the present dataset, it raises the possibility that broader reporting and recording gaps affected its completeness. More generally, passive and semi-structured pharmacovigilance processes may capture only a fraction of adverse events (Kessler, 1993[22]; Kostoff et al., 2020[23]; Lazarus, 2010[24]).

Third, the source file did not include a universal patient identifier, so duplicate records could not be excluded with complete certainty. One hospitalization record was explicitly marked in an original Excel comment as a duplicate and was excluded from analysis, while retained in Supplementary Data File 1. The comment was authored by Noa Cedar, identified in the file metadata as its creator and as having performed the active data collection for the subsequently published national myocarditis study (Mevorach et al., 2021[26]), suggesting that the file underwent at least some case-level review. No comparable duplicate annotation appears elsewhere, although additional overlap cannot be ruled out. The two worksheets were also cross-checked, and one death appearing in both was counted only once.

Fourth, the dataset does not specify the diagnostic criteria used for myocarditis or pericarditis, nor whether or how the reported diagnoses were clinically adjudicated. Diagnostic classifications are therefore presented as recorded and were derived from explicit terminology in the designated diagnosis, outcome, or clinical-narrative fields. Although the sensitivity analysis produced closely similar results when classification was restricted to the designated diagnostic fields, some degree of diagnostic or coding misclassification remains possible. Several source fields also contained missing, ambiguous, or implausible values, which were retained or treated as missing as documented in the *Supplementary Data File*. In addition, potentially identifying free-text medical histories and clinical narratives were removed from the supplementary dataset to protect confidentiality, limiting independent re-examination of some narrative-based classifications.

Finally, the subgroup comparisons were exploratory and were conducted within a potentially selected set of reported cases. The resulting associations therefore characterize the internal distribution of the reports and do not demonstrate that particular demographic or medical-history groups faced greater population-level risk. Similarly, the selected clinical narratives are illustrative and should not be interpreted as independent evidence of causality or frequency. The study's primary focus on myocarditis and pericarditis may also underrepresent the broader spectrum of serious adverse events documented in the dataset.

## Conclusions

Despite the limitations above, the disclosure of this previously inaccessible dataset (Supplementary Data File 1) and its analysis in the present study are of substantial public and scientific importance. The analysis revealed a notable concentration of reported cases of myocarditis and pericarditis, particularly among young individuals, including many for whom no meaningful medical history was identified. These cases were documented and reported to Pfizer early in the vaccination campaign, and the pattern observed in the dataset is consistent with the contemporaneous warning raised by the Israeli Ministry of Health, discussed in the Introduction, regarding a “large number” of such cases among young individuals (CDC, 2023, p. 668[6]).

Viewed in conjunction with contemporaneous regulatory communications and scientific publications, these findings point to two related concerns: (1) delays in communicating potentially important safety information and (2) limitations in how reported adverse events were publicly characterized. Together, these issues may have implications for clinical awareness, risk communication, and informed consent, with potential downstream effects on patient outcomes and public trust in pharmacovigilance processes.

## Declaration

### Author contributions

Literature Search: YO and YSR; Data Access and Verification: YO and YSR independently accessed the anonymized dataset and verified the underlying data reported in the manuscript; Data Acquisition: YSR (obtained the dataset); Data Curation: YO and YSR; Formal Analysis: YO and YSR; Methodology: YO; Investigation (including literature review and contextual analysis): YO and YSR; Study Design: YO with input from YSR; Writing - Original Draft: YO; Writing - Review & Editing: YSR and YO; Supervision: YO; Both authors have read and approved the final manuscript.

### Conflicts of interest

The authors declare no conflicts of interest.

### Funding

The authors received no funding for this work.

### Availability of data and materials

The anonymized case-level dataset is provided as Supplementary Data File 1. Additional contextual evidence is summarized in Supplementary Table 1.

### Ethics

This study was approved by the Institutional Ethics Committee of Ariel University (Approval No.: AU-SOC-YO-20251113).

### Artificial Intelligence (AI) - assisted technology

Artificial intelligence tools were used for limited language editing purposes only. The authors take full responsibility for all scientific content, analyses, and conclusions presented in the manuscript.

## Supplementary Material

Supplementary data

Supplementary information

## Figures and Tables

**Table 1:**
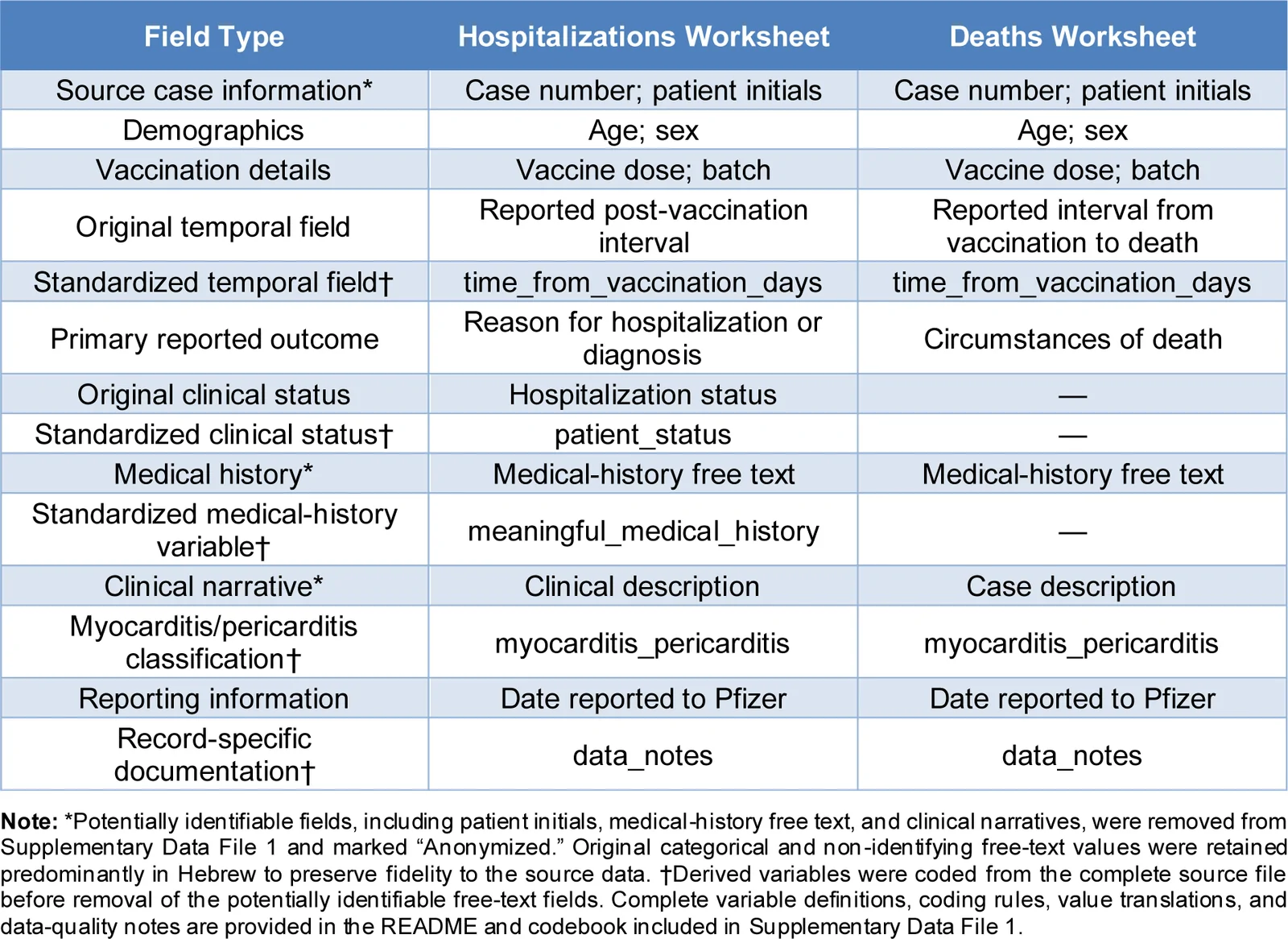
Original and derived data fields included in Supplementary Data File 1

**Table 2:**
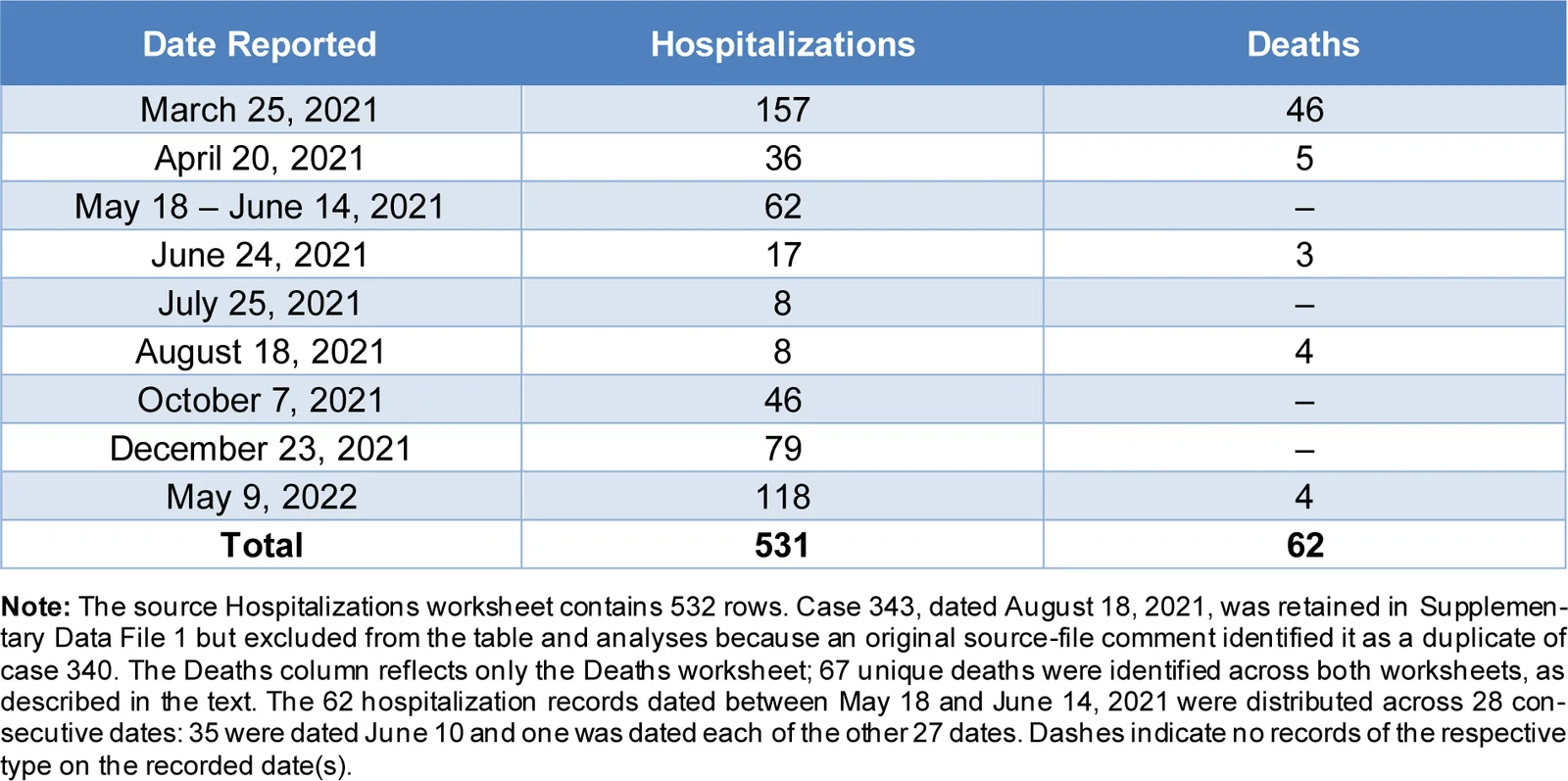
Chronological summary of records reported to Pfizer

**Table 3:**
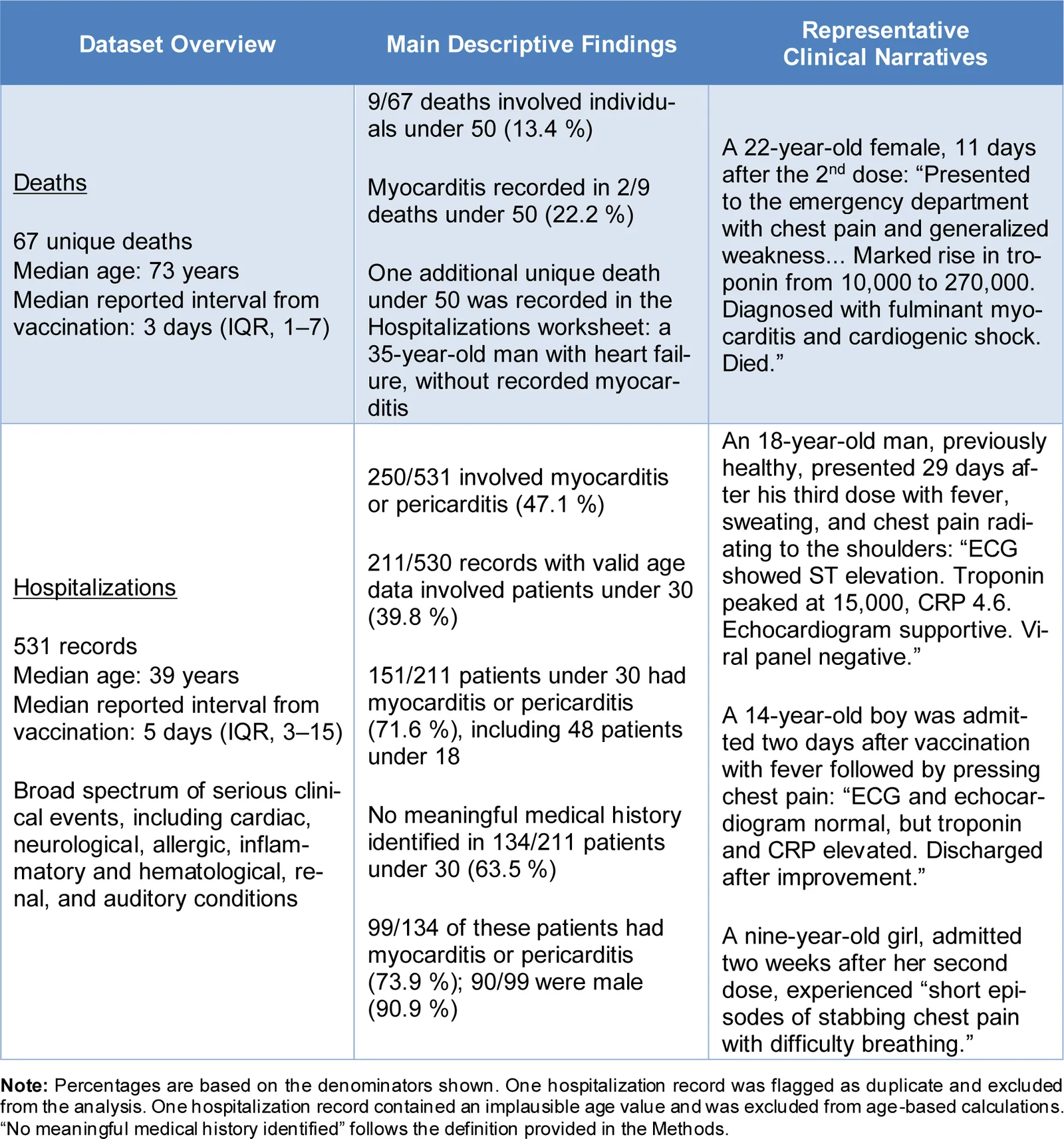
Overview and main findings of the pharmacovigilance dataset

**Table 4:**
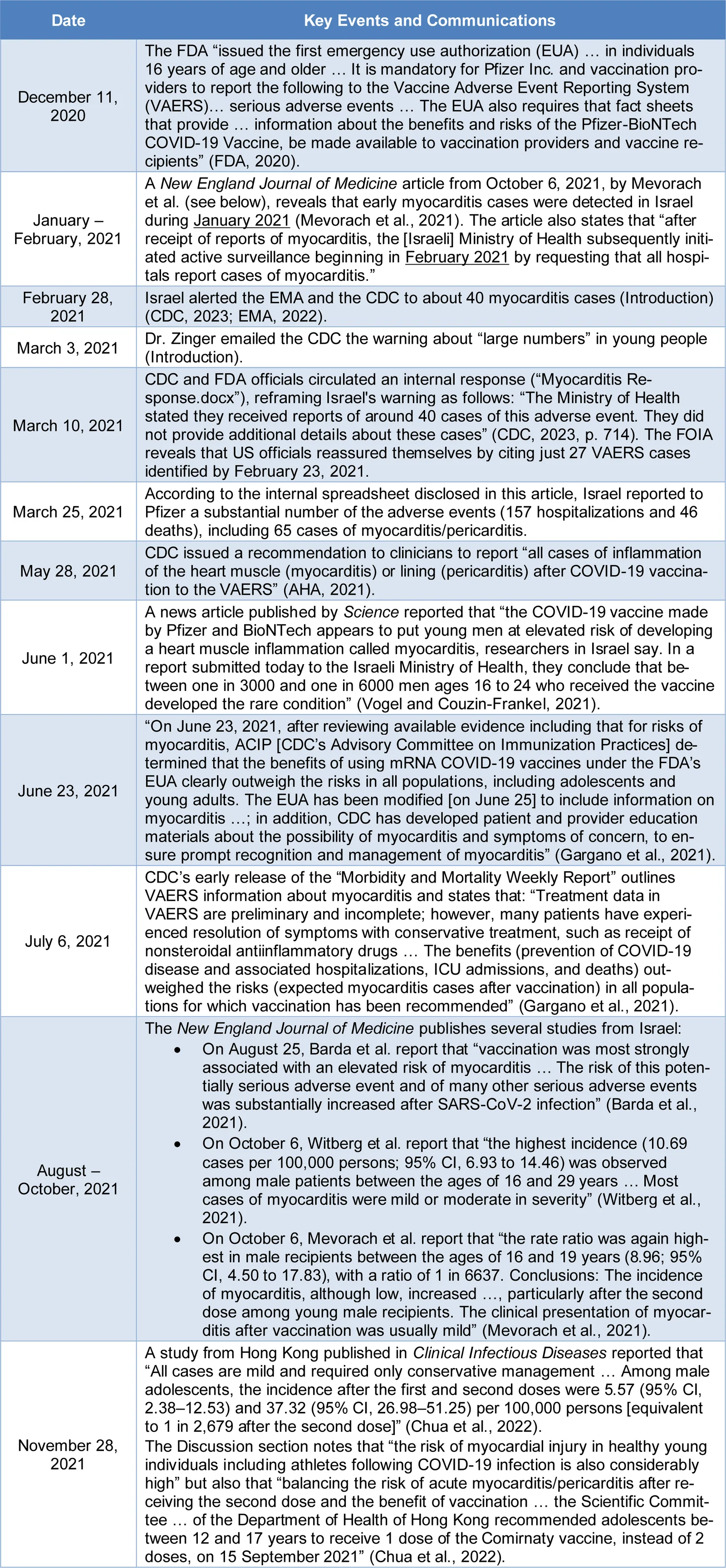
Timeline of the myocarditis safety signal communication

**Figure 1:**
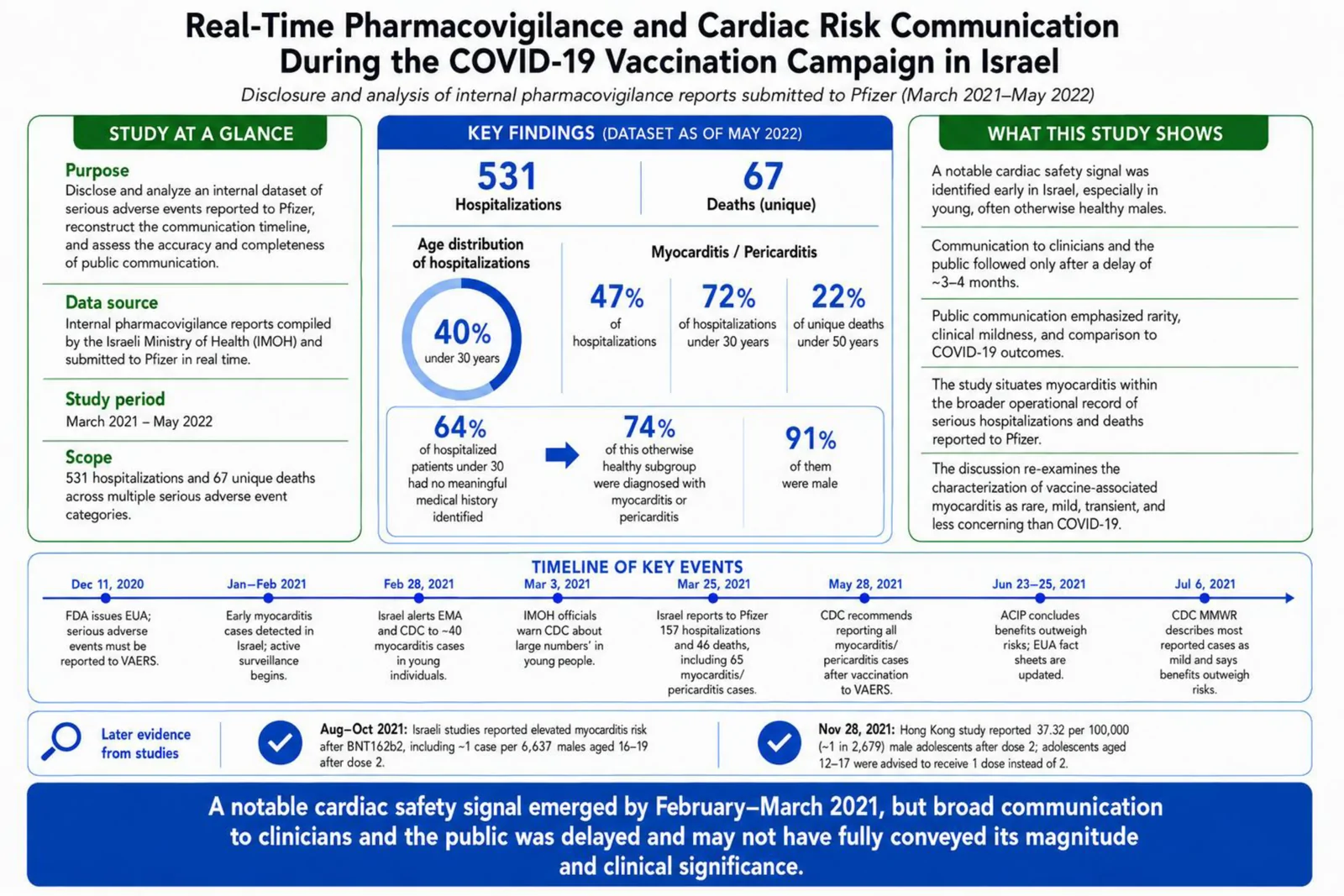
Graphical abstract
