# Supplementary material for: Real-time pharmacovigilance and cardiac risk communication during the COVID-19 vaccination campaign in Israel
Source: EXCLI J. 2026 Sep 3;25:1437–57. doi: 10.17179/excli2026-9596 (PMC13639178; doi:10.17179/excli2026-9596)
Supplement: Supplementary file 1 [file EXCLI-25-1437-s-002.pdf]

EXCLI Journal 2026;25:1437-1457 – ISSN 1611-2156
Received: June 08, 2026, accepted: August 09, 2026, published: September 03, 2026

Supplementary information to:

Original article:

REAL-TIME PHARMACOVIGILANCE AND CARDIAC RISK COMMUNICATION DURING THE COVID-19 VACCINATION CAMPAIGN IN ISRAEL

Yaakov Ophir[1,2*], Yaffa Shir-Raz[3]

[1] Ariel University, Ariel, Israel
[2] University of Cambridge, Cambridge, UK
[3] University of Haifa, Haifa, Israel

* **Corresponding author:** Dr. Yaakov Ophir, Ariel University, Ariel, Israel; University of Cambridge, Cambridge, UK; E-mail: yaakovophir@gmail.com

https://dx.doi.org/10.17179/excli2026-9596

This is an Open Access article distributed under the terms of the Creative Commons Attribution License (https://creativecommons.org/licenses/by/4.0/).

Supplementary Table 1: Accumulating evidence on clinical outcomes and pathophysiological mechanisms in COVID-19 vaccine–associated myocarditis. The evidence is drawn from cohort studies, imaging follow-up investigations, autopsy reports, systematic reviews, and mechanistic studies published between 2022 and 2025. To minimize interpretive bias, the findings are presented using direct quotations or closely abridged excerpts from the original publications.

Date	Study Details and Key Findings (Authors' Original Wording)
2022	*Tropical Medicine and Infectious Disease* (Mansanguan et al., 2022)<br>A prospective cohort study on Thai adolescents aged 13–18 reported that "cardiovascular manifestations were found in 29.24% of patients, ranging from tachycardia or palpitation to myopericarditis."<br><br>*The Journal of Pediatrics* (Schauer et al., 2022)<br>"In this cohort of adolescents aged 12-17 years with myopericarditis after the second dose … many had persistent cardiac magnetic resonance imaging findings at 3- to 8-month follow-up, including late gadolinium enhancement (LGE), which reflects myocardial fibrosis and scarring."<br><br>*Archives of Pathology & Laboratory Medicine* (Gill et al., 2022)<br>"The results of autopsies for 2 teenage boys who were found dead in bed 3 and 4 days after receiving the second dose of the Pfizer-BioNTech COVID-19 … revealed features resembling a catecholamine-induced injury … The myocardial injury seen in these postvaccine hearts is different from typical myocarditis and has an appearance most closely resembling a catecholamine-mediated stress (toxic) cardiomyopathy."

EXCLI Journal 2026;25:1437-1457 – ISSN 1611-2156
Received: June 08, 2026, accepted: August 09, 2026, published: September 03, 2026

Date	Study Details and Key Findings (Authors' Original Wording)
**2023**	*European Journal of Heart Failure* (Buergin et al., 2023)<br>A study on hospital employees reported that "mRNA-1273 booster vaccination-associated elevation of markers of myocardial injury occurred in about one out of 35 persons (2.8%), a greater incidence than estimated in meta-analyses of hospitalized cases with myocarditis."<br><br>*American Journal of Preventive Medicine* (Gao et al., 2023)<br>A systematic literature review indicated "that a higher incidence of myocarditis or pericarditis was found after COVID-19 vaccination. In addition, the risk of developing myocarditis or pericarditis was greater after the second dose than after the first dose."<br><br>*European Heart Journal* (Cho et al., 2023)<br>"A comprehensive nationwide study [from Korea] on the incidence and outcomes of COVID-19 vaccination-related myocarditis (VRM)" found that "sudden cardiac death [SCD] was the most serious and worrisome adverse reaction of COVID-19 vaccination … In eight SCD cases, VRM was not suspected as a clinical diagnosis or a cause of death before performing an autopsy".
**2024**	*Infectious Diseases* (Samimisedeh et al., 2024)<br>According to a systematic review, "on follow-up cardiac MRI, the persistence of LGE was observed in 76% (95% CI: 62 to 85%), but its extension declined compared to the baseline in almost all patients. Persistent LGE was accompanied by myocardial oedema in six patients, and it was consistent with myocardial fibrosis (LGE without oedema) in the remaining cases."<br><br>*eClinicalMedicine* (Jain et al., 2024)<br>"333 patients with C-VAM [COVID-19 vaccine-associated myocarditis] were compared with 100 patients with multisystem inflammatory syndrome in children... The primary outcome was presence of myocardial injury as evidenced by late gadolinium enhancement (LGE) … Mid-term clinical outcomes of C-VAM at a median follow-up of 178 days (IQR 114–285 days) were reassuring … [but] LGE persisted in 60% of the patients at follow up."<br><br>*ESC Heart Failure* (Hulscher et al., 2025)<br>"A systematic review of all published autopsy reports involving COVID-19 vaccination-induced myocarditis through 3 July 2023 [yielded] 14 papers that contained 28 autopsy cases … All 28 deaths were most likely causally linked to COVID-19 vaccination by independent review of the clinical information presented in each paper."

Date	Study Details and Key Findings (Authors' Original Wording)
2025	*Open Heart* (Warren et al., 2025)<br>"Long-term myocardial fibrosis is a common finding in patients following a diagnosis of COVID-19 vaccine-associated myocarditis."<br><br>*npj Vaccines* (Deng et al., 2025)<br>"Of 256 individuals who consented to follow up, mostly males following a second dose, 60% (133/221) had ongoing symptoms at 3-6 months and 35% (81/231) at 12-18 months."<br><br>*European Heart Journal* (Ramadan et al., 2025)<br>"More than two years after admission for COVID-19 vaccine-associated myocarditis, patients exhibit impaired left ventricular function but preserved right ventricular function."<br><br>JACC: *Case Reports* (Koizumi and Ono, 2025)<br>"Multiple micro-scars (MMS) found in the myocardium of 3 patients who died of unexplained cardiac arrest … All 3 patients had histories of COVID-19 booster vaccination, and 1 of the 3 patients had a history of COVID-19."<br><br>*Circulation* (Fanti et al., 2025)<br>"AMP [acute myopericarditis] development after mRNA vaccines is mediated by distinct immune components, including molecular mimicry, T-cell receptor affinity, and, importantly, homing imprinting"<br><br>*Science Translational Medicine* (Cao et al., 2025)<br>"Through analysis of human plasma data and in vitro experiments, we identified increased CXCL10 and IFN-γ after exposure to BNT162b2 or mRNA-1273 … Neutralization of CXCL10 and IFN-γ reduced vaccine-induced cardiac injury … These findings implicate CXCL10–IFN-γ signaling as a contributor to myocardial injury in experimental models of mRNA vaccination."

REFERENCES

Buergin N, Lopez-Ayala P, Hirsiger JR, Mueller P, Median D, Glarner N, et al. Sex-specific differences in myocardial injury incidence after COVID-19 mRNA-1273 booster vaccination. Eur J Heart Fail. 2023;25: 1871–1881.
https://doi.org/10.1002/ejhf.2978

Cao X, Manhas A, Chen YI, Caudal A, Mondejar-Parreño G, Zhu W, et al. Inhibition of CXCL10 and IFN-γ ameliorates myocarditis in preclinical models of SARS-CoV-2 mRNA vaccination. Sci Transl Med. 2025;17:eadq0143.
https://doi.org/10.1126/scitranslmed.adq0143

Cho JY, Kim KH, Lee N, Cho SH, Kim SY, Kim EK, et al. COVID-19 vaccination-related myocarditis: a Korean nationwide study. Eur Heart J. 2023;44:2234–2243.
https://doi.org/10.1093/eurheartj/ehad339

Deng L, Van Eldik A, O'Moore M, Buttery J, Cheung A, Cox N, et al. Surveillance and follow up outcomes of myocarditis after mRNA COVID-19 vaccination in Australia. NPJ Vaccines. 2025;10:155.
https://doi.org/10.1038/s41541-025-01206-w

Fanti S, Dyer C, Ingimarsdóttir IJ, Harding D, Wang G, D'Amati A, et al. Combined adaptive immune mechanisms mediate cardiac injury after COVID-19 vaccination. Circulation. 2025;152:1485–1500.
https://doi.org/10.1161/CIRCULATIONAHA.125.074644

Gao J, Feng L, Li Y, Lowe S, Guo Z, Bentley R, et al. A systematic review and meta-analysis of the association between SARS-CoV-2 vaccination and myocarditis or pericarditis. Am J Prev Med. 2023;64:275–284.
https://doi.org/10.1016/j.amepre.2022.09.002

Gill JR, Tashjian R, Duncanson E. Autopsy histopathologic cardiac findings in 2 adolescents following the second COVID-19 vaccine dose. Arch Pathol Lab Med. 2022;146:925–929.
https://doi.org/10.5858/arpa.2021-0435-SA

Hulscher N, Hodkinson R, Makis W, McCullough PA. Autopsy findings in cases of fatal COVID-19 vaccine-induced myocarditis. ESC Heart Fail. 2025;12:3212–3225.
https://doi.org/10.1002/ehf2.14680

Jain SS, Anderson SA, Steele JM, Wilson HC, Muniz JC, Soslow JH, et al. Cardiac manifestations and outcomes of COVID-19 vaccine-associated myocarditis in the young in the USA: longitudinal results from the Myocarditis After COVID Vaccination (MACiV) multicenter study. EClinicalMedicine. 2024;76:102809.
https://doi.org/10.1016/j.eclinm.2024.102809

Koizumi T, Ono M. Cardiac multiple micro-scars: an autopsy study. JACC Case Rep. 2025;30:103083.
https://doi.org/10.1016/j.jaccas.2024.103083

Mansanguan S, Charunwatthana P, Piyaphanee W, Dechkhajorn W, Poolcharoen A, Mansanguan C. Cardiovascular manifestation of the BNT162b2 mRNA COVID-19 vaccine in adolescents. Trop Med Infect Dis. 2022;7:196.
https://doi.org/10.3390/tropicalmed7080196

Ramadan MN, Soeskov Davidovski F, Espersen C, Al-Rubai AH, Khoraizat A, Johansen ND, et al. Long term cardiac function and symptoms in patients following COVID-19 vaccination myocarditis. Eur Heart J. 2025; 46:ehaf784.2583.
https://doi.org/10.1093/eurheartj/ehaf784.2583

Samimisedeh P, Jafari Afshar E, Tayebi A, Rastad H. Post-acute midterm follow-up cardiac MRI findings and clinical outcomes in patients with COVID-19 vaccine-associated myocarditis: a comprehensive systematic review and meta-analysis. Infect Dis (Lond). 2024; 56:193–205.
https://doi.org/10.1080/23744235.2023.2286289

Schauer J, Buddhe S, Gulhane A, Sagiv E, Studer M, Colyer J, et al. Persistent cardiac magnetic resonance imaging findings in a cohort of adolescents with post-coronavirus disease 2019 mRNA vaccine myopericarditis. J Pediatr. 2022;245:233–237.
https://doi.org/10.1016/j.jpeds.2022.03.032

Warren J, Cheng D, Crawford NW, Jones B, Ng RL, Alafaci A, et al. Improved diagnosis of COVID-19 vaccine-associated myocarditis with cardiac scarring identified by cardiac magnetic resonance imaging. Open Heart. 2025;12:e003333.
https://doi.org/10.1136/openhrt-2025-003333
